# Association between polymorphisms of *FCRL3,* a non-HLA gene, and Behçet’s disease in a Chinese population with ophthalmic manifestations

**Published:** 2008-11-28

**Authors:** Ke Li, Min Zhao, Shengping Hou, Liping Du, Aize Kijlstra, Peizeng Yang

**Affiliations:** 1 First Affiliated Hospital of Chongqing Medical University, Chongqing, P.R. China; 2 Zhongshan Ophthalmic Center, Sun Yat-sen University, Guangzhou, P.R. China; 3 Eye Research Institute Maastricht, Department of Ophthalmology, University Hospital Maastricht, Maastricht, the Netherlands

## Abstract

**Purpose:**

Studies have shown a strong association of human leukocyte antigens-B51 (*HLA-B51*) with Behçet’s disease (BD). However, little is known about the association of non-*HLA* genes with BD. The polymorphisms of the Fc receptor-like 3 gene (*FCRL3*), −169C/T, −110A/G, +358C/G, and +1381A/G, have been reported to be associated with several autoimmune diseases. This study was designed to determine whether the polymorphisms of *FCRL3* were associated with susceptibility to BD in a Chinese population mainly with ocular involvement.

**Methods:**

A case-control study was performed in 245 Chinese BD patients and 289 controls. Four single nucleotide polymorphisms (SNPs; −169C/T, −110A/G, +358C/G, and +1381A/G) in *FCRL3* were detected using polymerase chain reaction restriction fragment length polymorphisms (PCR-RFLP). *HLA-B51* genotyping was performed by the PCR sequence specific primers method as described previously.

**Results:**

The results showed a significantly higher frequency of the G allele at the −110 site in BD patients compared with that in controls (corrected p=0.044, 75.3% versus 67.5%, χ^2^=7.72). Haplotype CGCG frequency was significantly higher in patients than in controls (corrected p=0.0096) whereas haplotype TACG frequency was significantly lower in patients compared with controls (corrected p=0.032). There was no relationship between clinical signs and *FCRL3* polymorphisms. No significant difference was observed between patients and controls after *HLA-B51* stratification concerning the four SNPs.

**Conclusions:**

Our study suggests that the −110 G allele and the haplotype CGCG of *FCRL3* are positively associated with BD in a Chinese population and that the haplotype ATCG might be a protective haplotype for BD.

## Introduction

Behçet’s disease (BD) is an idiopathic, multisystem, recurrent chronic inflammatory disease in China. The major clinical manifestations include recurrent oral and genital ulceration, uveitis, and erythema nodosum. Panuveitis was the most common type of uveitis in BD patients in China, although solo anterior uveitis is observed in certain patients [1]. BD exists worldwide and has significant regional differences. It is quite common along the ancient ‘Silk Road’ countries extending from China to the Mediterranean area [2]. Although the precise etiology of BD remains unknown, extensive studies suggest that autoimmunity and genetic factors are involved in its pathogenesis. It has been shown that polymorphisms of several genes such as human leukocyte antigens-B51 (*HLA-B51*), intercellular adhesion molecule-1 (*ICAM-1*) [3], and tumor necrosis factor–α (*TNF–α*) [4] are associated with susceptibility to BD. Among these genes, *HLA-B51* is the most strongly associated gene with BD in different ethnic populations [5-8], particularly those from the Middle East to the Far East [9], coinciding with the distribution of BD.

Fc receptor-like genes (*FCRLs*), also known as *FCRHs* (Fc receptor homology) or *IRTAs* (immunoglobulin superfamily receptor translocation associated genes) [10], and *SPAPs* (SH2 domain containing phosphatase anchor proteins) cluster on human chromosome 1q21–23 adjacent to the Fc receptor genes [11]. *FCRLs* have a high structural homology with the classical Fcγ receptor genes (*FcγR*) and contain six Ig superfamily members that are known as *FCRL1–FCRL6* according to their chromosomal order [12]. Recently, a classical study by Kochi et al. [13] in Japan found that four single nucleotide polymorphisms (SNPs) of *FCRL3* (−169C/T, −110A/G, +358C/G, and +1381A/G) were associated with rheumatoid arthritis (RA) and that SNP rs7528684C/T was associated with autoimmune thyroid disease (AITD) and systemic lupus erythematosus (SLE). As various autoimmune disorders may share common pathogenic pathways, genes that have been found to be involved in one autoimmune disease may also be considered as a candidate associated with other autoimmune diseases. Until now, a *FCRL3* polymorphism has yet to be investigated in BD. Therefore, this study was designed to investigate the association of the aforementioned four SNPs of *FCRL3* (−169C/T, −110A/G, +358C/G, and +1381A/G) with susceptibility to BD in a Chinese population, mainly with ocular involvement. The result showed a positive association of the −110G allele and the haplotype CGCG and a negative association of the haplotype TACG with BD.

## Methods

### Study participants

Patients and controls were recruited from the Zhongshan Ophthalmic Center of Sun Yat-sen University (Guangzhou, P.R. China) and the First Affiliated Hospital of Chongqing Medical University (Chongqing, P.R. China). The test subjects consisted of 245 Chinese BD patients (aged 28.6±6.0 years) and 289 healthy controls (aged 34.2±10.0 years). All control subjects were matched ethnically and geographically with the patients.

The diagnosis of Behçet’s disease followed the criteria of the International Study Group for Behçet’s disease [14]. The clinical characteristics of the patients are presented in Table 1. All subjects gave their written informed consent for this study, and the study protocol was approved by the local institutional ethics committee.

**Table 1:** Clinical characteristics of patients with BD.

**Clinical features**	**Patients (n=245)**	
	**N**	**%**
Male	204	83.3
Female	41	16.7
Uveitis	235	95.9
Hypopyon	53	21.6
Oral ulcer	226	92.2
Skin lesions	120	49
Genital ulcer	95	38.8
Positive pathergy	81	33.1
Arthritis	60	24.5

N=number of patients.

### Single nucleotide polymorphisms and genotyping

DNA was prepared by proteinase K digestion and salt extraction from peripheral blood of patients and controls and stored at −70 °C until use. The four SNPs in *FCRL3*, namely −169C/T (rs7528684 or fcrl3_3), −110G/A (rs11264799 or fcrl3_4), +358 C/G (rs945635 or fcrl3_5), and +1381A/G (rs3761959 or fcrl3_6), were genotyped by polymerase chain reaction restriction fragment length polymorphism (PCR-RFLP) .

Genotyping of the −110A/G SNP was performed according to the method as described previously [15]. The primers of the three remaining sites were designed using Primer Premier 5.0 software (Premier Biosoft International, Palo Alto, CA). The details of the primers and enzymes used for PCR-RFLP genotyping are presented in Table 2.

**Table 2:** Details of the primers and enzymes used for PCR-RFLP genotyping.

**SNPs**	**dbSNP ID**	**Forward primer**	**Reverse primer**	**Tm (°C)**	**Restriction enzyme**
−169C/T(fcrl3_3)	rs7528684	GGAAAATAATACA AATGTACAGATTA	GGCTTTAAAA AACGGTGGTAC	56.9	BsmFI
−110A/G(fcrl3_4)	rs11264799	CTCAATCCCGGT AGTGATACA	CTCATAACAAC TTATGTGAGA	56.9	Ple I
+358C/G(fcrl3_5)	rs945635	TTATAGCCCATCTA CTCACTCAGGATCA	CCGGGATTGAGA TACAAACAGCATTT	60.3	HaeIII
+1381A/G(fcrl3_6)	rs3761959	TCCGACTTTTTCA GTCTCTAGGTTTT	TGATAGCAGCACTA GCTTGGACATTCA	60.3	MspI

Tm=melting temperature

PCR was performed in 15 μl volumes containing 7.5 μl Premix Taq (Ex Taq Version; TaKaRa Biotechnology Co. Ltd, Dalian, China), 0.5 μl primers (10 μmol/l), and 0.1 μg of genomic DNA. The PCR products were then digested by the proper restriction enzymes and separated by electrophoresis on 2~3% agarose gels and stained with GoldView™ (SBS Genetech, Beijing, China). The images were recorded digitally. *HLA-B51* genotyping was performed by the PCR-sequence specific primers (PCR-SSP) method as described previously [16].

### Statistical analysis

Statistical analysis was performed with the SPSS version 12.0 for Windows (SPSS Inc., Chicago, IL). Hardy–Weinberg equilibrium (HWE) was tested by the χ^2^ test. We evaluated the frequency of genotypes and alleles in this study using the χ^2^ test or Fisher’s exact test. The haplotype frequency and linkage disequilibrium (LD) of the SNPs were estimated with the Haploview 3.2 program [17]. A haplotype frequency less than 0.03 was not studied further. All the data were corrected by Bonferroni correction.

## Results

**Figure 1:**
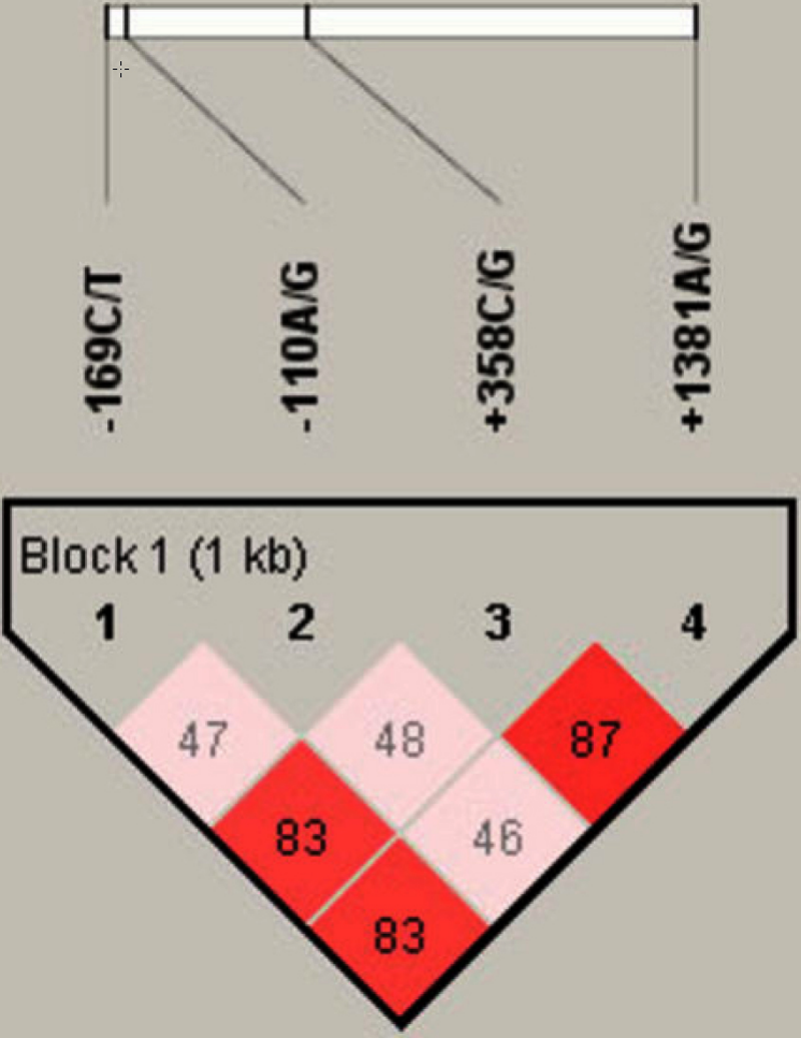
Location and pair-wise linkage disequilibrium values of four *FCRL3* SNPs in a Chinese population. These four SNPs span 1 kb of the *FCRL3* region. Values of the pair-wise D’ (multiply by 100) are shown in one block.

Four SNPs in *FCRL3* were determined in 245 BD patients and 289 healthy controls. The results showed that all the cases and controls were in HWE. We analyzed LD with these four SNPs and haplotype analysis with Haploview software, and the four SNPs of *FCLR3* are not in high LD (D’=46–87; r^2^=0.10–0.74; Figure 1). The frequency of the G allele at the −110 site was significantly higher in patients (75.3%) than in controls (67.5%; corrected p=0.044, χ^2^=7.72; Table 3). The −110 GG genotype was also found to be increased in patients (p=0.007), but this significance was lost after Bonferroni correction (corrected p=0.084). The frequency of the −169C:−110G:+358C:+1381G haplotype was significantly higher in patients compared with controls (13.3% versus 7.0%, respectively; p=6.0×10^−4^, corrected p=0.0096, χ^2^=11.70; Table 4). The frequency of haplotype −169T:−110A:+358C:+1381G was significantly lower in patients than that in controls (3.0% versus 7.0%, respectively; p=0.002, corrected p=0.032, χ^2^=9.27; Table 4). No significant difference in the remaining three SNPs tested was observed between BD patients and controls in the distribution of other alleles and genotypes.

**Table 3:** Frequency of alleles and genotypes of four *FCRL3* SNPs in BD patients and controls.

**SNPs**	**Genotype/ allele**	**BD (%)**	**Controls (%)**	**χ^2^**	**p**	**Corrected p**	**OR (95% CI)**
−169 C→T	CC	69 (28.3)	86 (29.8)	1.95	0.38	NS	
	CT	121 (49.6)	127 (43.9)				
	TT	54 (22.1)	76 (26.3)				
	C	259 (53.3)	299 (51.7)	0.26	0.61	NS	1.1 (0.8–1.3)
	T	227 (46.7)	279 (48.3)				0.9 (0.7–1.2)
−110 A→G	AA	17 (7.1)	46 (16.0)	10.03	0.007	NS	
	AG	85 (35.3)	95 (33.0)				
	GG	139 (57.7)	147 (51.0)				
	A	119 (24.7)	187 (32.5)	7.72	0.0055	0.044	0.7 (0.5–0.9)
	G	363 (75.3)	389 (67.5)				1.5 (1.1–1.9)
358 C→G	CC	81 (33.2)	97 (33.6)	1.12	0.57	NS	
	CG	122 (50.0)	134 (46.4)				
	GG	41 (16.8)	58 (20.1)				
	C	283 (58.0)	328 (56.7)	0.17	0.68	NS	1.1(0.8–1.4)
	G	205 (42.0)	250 (43.3)				0.9(0.7–1.2)
1381 A→G	AA	40 (16.4)	54 (18.8)	0.5	0.78	NS	
	AG	122 (50.0)	140 (48.6)				
	GG	82 (33.6)	94 (32.6)				
	A	203 (58.4)	248 (56.9)	0.23	0.63	NS	0.9 (0.7–1.2)
	G	285 (41.6)	328 (43.1)				1.1 (0.8–1.4)

BD= Behçet’s disease; OR=odds ratio; 95% CI=95% confidence interval; NS=not significant.

**Table 4:** Haplotype frequencies in BD patients and controls.

**Haplotypes**	**BD n=245 (%)**	**Controls n=289 (%)**	**χ^2^**	**Fisher’s p**	**Corrected p**	**OR**
TGCG	180.4 (37.0)	210.5 (36.4)	0.033	0.86	NS	1.1 (0.9–1.5)
CAGA	89.0 (18.2)	116.0 (20.1)	0.57	0.45	NS	1.0 (0.8–1.4)
CGGA	84.2 (17.2)	106.9 (18.5)	0.28	0.6	NS	0.8 (0.6–1.1)
CGCG	64.9 (13.3)	40.6 (7.0)	11.7	0.0006	0.0096	2.4 (1.6–3.7)
TACG	14.5 (3.0)	41.3 (7.0)	9.27	0.002	0.032	0.3 (0.1–0.6)

BD=Behçet’s disease; OR=odds ratio; NS=not significant.

Since the BD patients showed different clinical features, we further analyzed the relationship between the SNPs and various clinical parameters such as uveitis, hypopyon, oral or genital ulcers, multiform skin lesions, and arthritis. No significant difference was noted between any of the mentioned clinical characteristics and the investigated SNPs. Of the 245 patients, 204 (83.3%) were men and 41 (16.7%) were women. No significant difference was found in the four SNPs between male and female patients.

As *HLA-B51* has been shown to be strongly associated with BD, we also investigated its frequency in patients and controls. The result showed that 99 (40.4%) patients were *HLA-B51* positive as compared to 28 (9.7%) of the controls, a significant difference. *HLA-B51* stratification analysis showed a significant difference for the −110 A/G allele, the AA genotype, and the haplotype CGCG and TACG. The difference, however, lost its significance when the Bonferroni correction was applied (Table 5).

**Table 5:** Frequency of alleles and genotypes at four *FCRL3* SNPs in *HLA-B51* negative BD patients and in *HLA-B51* negative controls.

**SNPs**	**Genotype/allele**	**BD n=146 (%)**	**Controls n=261 (%)**	**χ^2^**	**p**	**Corrected p**	**OR (95% CI)**
−169 C→T	CC	45 (30.8)	77 (29.5)	0.22	0.9	NS	
	CT	67 (45.9)	118 (45.2)				
	TT	34 (23.3)	66 (25.3)				
	C	159 (54.1)	272 (52.1)	0.29	0.59	NS	1.1 (0.8–1.4)
	T	135 (45.9)	250 (47.9)				0.9 (0.7–1.2)
−110 A→G	AA	10 (6.9)	43 (16.5)	7.73	0.02	NS	
	AG	56 (38.6)	86 (33.1)				
	GG	79 (54.5)	131 (50.4)				
	A	76 (26.0)	172 (33.1)	4.38	0.04	NS	0.7 (0.5–1.0)
	G	216 (74.0)	348 (66.9)				1.4 (1.0–1.9)
+358 C→G	CC	47 (32.2)	85 (32.6)	0.24	0.89	NS	
	CG	73 (50.0)	125 (47.9)				
	GG	26 (17.8)	51 (19.5)				
	C	168 (57.1)	295 (56.5)	0.03	0.86	NS	1.0 (0.8–1.4)
	G	126 (42.9)	227 (43.5)				1.0 (0.7–1.3)
+1381 A→G	AA	26 (17.8)	49 (18.8)	0.5	0.78	NS	
	AG	72 (49.3)	127 (48.8)				
	GG	48 (32.9)	84 (32.3)				
	A	125 (42.5)	225 (43.3)	0.043	0.84	NS	1.0 (0.7–1.3)
	G	169 (57.5)	295 (56.7)				1.0 (0.8–1.4)

BD= Behçet’s disease; OR=odds ratio; 95% CI=95% confidence interval; NS= not significant.

## Discussion

In this study, we analyzed the association of four *FCRL3* SNPs with BD in a Chinese population. The results showed that the −110G allele was significantly increased in the BD patients. The frequency of the haplotype CGCG was also found to be significantly increased in these patients whereas a significantly decreased frequency of haplotype TACG was noted. These results suggest that polymorphisms of *FCRL3* are associated with the susceptibility to BD in a Chinese population.

BD is one of the most common uveitis entities in China [1]. Its distribution along the old ‘Silk Road’ and the clustering of the disease in families suggest that genetic factors may play a role in the pathogenesis of this disease. *HLA-B51* has been shown to be strongly associated with BD in different ethnic populations. Little is known about the association of non-*HLA* genes with BD, although polymorphisms of several genes have been investigated in this disease [3,4]. In this study, we focused on the association of four *FCRL3* SNPs with BD mainly because of the role this gene may play in the pathogenesis of autoimmunity and its association with other autoimmune diseases. *FCRL* have been shown to have a high structural homology with the classical *FcγR*, which are expressed mainly on the surface of a variety of immune cells. Moreover, previous studies showed that polymorphisms of *FCγR* were associated with a variety of autoimmune diseases [18,19]. *FCRL* has been found to be expressed predominantly on B cells and has also been detected at low levels in CD4+ and CD8+ T cells [13]. *FCRL3* may play a role in the differentiation of B cells into autoreactive cells and has been presumed to function through modulating signal transduction via activation/inactivation of signaling tyrosine protein kinases [20]. Furthermore, the −169C/T SNP of *FCRL3* has been shown to influence the level of *FCRL3* expression on B cells through altering the binding affinity of the nuclear factor-kappa-B (NF-κB) [13], an important immunoregulatory factor, according to recent studies in both murine models and humans with diverse forms of autoimmunity [21]. It is not clear whether the −110 SNP has any functional role. The association of this SNP with BD as disclosed in our study appears to suggest a possible involvement of *FCRL3* in the development of BD.

Our study showed a significantly higher frequency of the G allele at the −110 site in BD patients, which is consistent with that observed in patients with autoimmune Addison’s disease (AAD) in a UK population [15]. This identical polymorphism between BD and AAD seemed to show a susceptibility that both have in common. Interestingly, two studies by Japanese investigators showed a decreased frequency of the G allele in the −110 site in RA and autoimmune pancreatitis [13,22]. Furthermore, a study reported by Simmonds et al. [23] showed that three of the four SNPs studied here (the −110 SNP was the exception) were associated with Graves’ disease.

With regard to the haplotype analysis, we found two haplotypes, CGCG and TACG, that were associated with BD. The former was found to be significantly higher in BD patients while the latter was lower in BD patients. The findings suggest that the *FCRL3* −110G allele may be linked with susceptibility to BD, and those people with the haplotype CGCG are more prone to BD than those without this haplotype. On the contrary, the haplotype ATCG might be a protective haplotype to BD. Our results were different from those found in AAD [15] and SLE [24]. In the study of AAD, seven alleles and haplotypes were investigated, the haplotype TGGGAAA was found to be significantly increased among AAD patients compared to the controls. In the study of SLE, the frequency of haplotype CGA was found to be significantly higher among SLE patients compared to the controls.

As BD shows a variety of manifestations clinically and the aforementioned result revealed an association of the polymorphisms of *FCRL3* with this disease, it is reasonable to test whether certain clinical features are linked to this polymorphism. Unexpectedly, none of the clinical features including uveitis, hypopyon, oral ulcer or genital ulcer, multiform skin lesions, and arthritis were found to be associated with the identified susceptible allele at the −110 site. Similarly, there was no association between any of the investigated clinical features and the haplotypes. These results are generally consistent with those in a previous study performed in our laboratory recently searching for an association between *SUMO4* polymorphisms and BD. This study found no association between haplotype AGAT, which was already found to be significantly lower in BD patients, and any of the clinical characteristics in BD patients [25]. However, it is worthwhile to point out that there was a bias in the recruited BD patients in our current study. All the patients enrolled in this study came from ophthalmic centers, and the results merely revealed the association of the *FCRL3* polymorphisms with BD patients, mainly with ocular involvement. The association of *FCRL3* polymorphisms with BD in the whole population with this disease should be studied on the patients from dermatology and rheumatology centers.

Previous studies have showed that the association of *FCRL3* with diseases could be more striking after stratification with some parameters [26,27]. Genetic susceptibility to BD is well documented for *HLA-B51*. We therefore analyzed the association of the −110 SNP and the remaining three SNPs with BD based on the *HLA-B51* antigen stratification. Unfortunately, we did not find any association of these SNPs and haplotypes with BD after stratification for *HLA-B51*. This could be due to the insufficient sample size. Our sample size for the −110 SNP (28 *HLA-B51* positive normal controls and 99 *HLA-B51* positive BD patients) can only reach a 26% power value to detect a 2.0 odds ratio (OR) value at the 5% significance level. As there is a substantial difference in clinical manifestations between male and female BD patients, a stratification analysis according to sex was also performed in our study. Similarly, there was also no association following sex stratification in these patients. The small size of the sample for the female BD patients may be insufficient for this analysis. Therefore, a larger patient population is needed to clarify the association of *FCRL3* with Behcet’s disease in different *HLA* and sex status.

In conclusion, our study revealed that the −110G allele and haplotype CGCG are positively associated and haplotype TACG is negatively associated with the susceptibility to BD. These results may provide clues for the development of an adequate and effective therapy [28]. A pharmacogenomic relationship has been studied in open-angle glaucoma between β1-adrenergic receptor with betaxolol, between prostaglandin F2α receptor and the latanoprost, and between glucocorticoid receptor and intraocular pressure [29]. It is important to test whether the polymorphisms revealed by our study are associated with the sensitivity of certain medicines in the treatment of BD. As a multi-systemic autoimmune disease, BD may share mechanisms in common with other autoimmune diseases. Therefore, the predisposing gene for BD may also be involved in other autoimmune diseases in a similar way. It is necessary to clarify whether the discovered polymorphisms of *FCRL3* are associated with other autoimmune diseases. Furthermore, studies are needed to investigate whether the observed associations with BD are also present in other ethnic populations.
